# Physiological and Perceptual Responses to Single-player vs. Multiplayer Exergaming

**DOI:** 10.3389/fspor.2022.903300

**Published:** 2022-06-16

**Authors:** Aarón Soria Campo, Alf Inge Wang, Trine Moholdt, Jonathan Berg

**Affiliations:** ^1^Faculty of Medicine and Health Sciences, Norwegian University of Science and Technology, Trondheim, Norway; ^2^Department of Computer Science, Faculty of Information Technology and Electrical Engineering, Norwegian University of Science and Technology, Trondheim, Norway; ^3^Department of Circulation and Medical Imaging, Faculty of Medicine and Health Sciences, Norwegian University of Science and Technology, Trondheim, Norway; ^4^St. Olav's University Hospital, Trondheim, Norway

**Keywords:** active video game, cardiorespiratory fitness, exercise training, fitness, gamification, health technology, persuasive technology

## Abstract

**Rationale:**

Since many modern exergames include a multiplayer component, this study aimed to compare the physiological and perceptual responses between playing a cycling exergame alone or with others.

**Methods:**

In this randomized crossover study, 15 healthy individuals aged between 10 and 30 years completed a single-player and a multiplayer exergaming session. The main outcomes were exercise intensity, measured as oxygen uptake (V°O_2_) and heart rate (HR), and perceived enjoyment, pleasure, and exertion.

**Results:**

Peak HR was significantly higher during multiplayer (172 ± 23 beats per minute [bpm]) vs. single-player exergaming (159 ± 27 bpm) with a mean difference of 13 bpm (95% CI: 2 to 24, *p* = 0.02). Peak V°O_2_ was 33.6 ± 9.5 mL·kg^−1^·min^−1^ and 30.4 ± 9.1 mL·kg^−1^·min^−1^ during multiplayer and single-player exergaming, respectively with no statistically significant difference between conditions (3.2, 95% CI: −0.2–6.6 mL·kg^−1^·min^−1^, *p* = 0.06). Average HR, average V°O_2_ and perceptual responses did not differ between single- and multiplayer exergaming.

**Conclusion:**

Other than inducing a higher HR, multiplayer exergaming showed no significant benefits on exercise intensity or perceptual responses over single-player exergaming. However, the higher peak HR and a tendency of higher peak V°O_2_ intensity during multiplayer exergaming imply that multiplayer exergaming may offer some advantages over single-player exergaming that could impact the potential health benefits of exergaming.

## Introduction

Higher levels of physical activity (PA), long-term PA, and recent increases in PA are all strongly associated with a decreased risk of cardiometabolic disease, several cardiometabolic risk factors, and mortality (Cleven et al., 2020; Leskinen et al., 2020; Moholdt et al., 2021). Despite these well-known health benefits of PA, adherence to current PA guidelines is poor and decreases with age. Whereas 90% of Norwegian 6-years old adhere to the PA guidelines, adherence rates are gradually reduced to only 48% in adolescents and 33% in adults (Hansen et al., 2019). Furthermore, due to social distancing measures and lockdowns, the COVID-19 pandemic has led to a further decrease in population PA levels (Castañeda-Babarro et al., 2020). Therefore, it is vital to explore exercise alternatives that could boost adherence to PA.

Enjoyment is an essential mediator for exercise and PA adherence (Rodrigues et al., 2020; Teixeira et al., 2020) and should be considered when exploring alternative exercise modes. Exergaming is the playing of digital games requiring physical effort from the user to affect a change in exercise and PA behavior (Baranowski et al., 2008; Oh and Yang, 2010). Exergaming aims to explore the general interest in digital gaming, and just like for exercise, enjoyment is a crucial factor for the continued play of digital games (Sweetser and Wyeth, 2005; Neys et al., 2014). Accordingly, exergaming can be a more enjoyable alternative to traditional PA and exercise (Moholdt et al., 2017; Martin-Niedecken et al., 2020). In addition to providing the users with a pleasant experience, an exergame should induce an exercise response that promotes beneficial physiological adaptations (Sinclair et al., 2007). Exercising with a vigorous intensity appears to be particularly important to accrue health benefits, especially in youths (Gralla et al., 2019). The superior health benefits from vigorous vs. moderate-intensity exercise may stem from its more robust effect on cardiorespiratory fitness (Gralla et al., 2019; Wagner et al., 2021). The time spent at a high relative intensity may be critical for improving cardiorespiratory fitness and health (Midgley and Mc Naughton, 2006). As such, lengthening the duration of bouts at a high intensity will prolong the time spent with a high relative exercise intensity (Midgley and Mc Naughton, 2006). Although most exergames only elicit light-to-moderate exercise intensities, some newly developed exergames hold promise by producing higher exercise intensities (Moholdt et al., 2017; Berg and Moholdt, 2020; Martin-Niedecken et al., 2020; Ketelhut et al., 2022).

Many digital games, exergames included, are played with others (multiplayer). Social interaction in games contributes to player enjoyment (Sweetser and Wyeth, 2005). Games like Pokémon Go have health and social impact on the player from the social features provided through the game (Wang and Skjervold, 2021). Still, few have examined how the presence of a co-player affects the attractiveness and effectiveness of exergaming. Since exergames have been proposed as a beneficial tool during social distancing and self-isolation, it is essential to compare single- and multiplayer exergaming (Viana et al., 2021). A recent study by Gorsic et al. (2020) demonstrated that enjoyment, interest, and exercise intensity were higher when playing an arm exergame together with a physical co-player than when playing against a computer-generated player (single-player). However, in that study, participants played an arm exergame primarily used for arm rehabilitation (Gorsic et al., 2020) which likely limits its potential to improve cardiorespiratory fitness and health.

The primary aim of this study was to compare the physiological and perceptual responses to single- vs. multiplayer exergaming. Our secondary aim was to explore if specific participant characteristics, exergaming skills, or exergaming characteristics could explain the physiological and perceptual responses. We hypothesized that exercise intensity, enjoyment, and pleasure would be higher in multiplayer vs. single-player exergaming. Furthermore, we hypothesized that the number of rounds played during each exergaming session would affect time spent with vigorous and high exercise intensity.

## Methods

### Study Design

**Figure 1:**
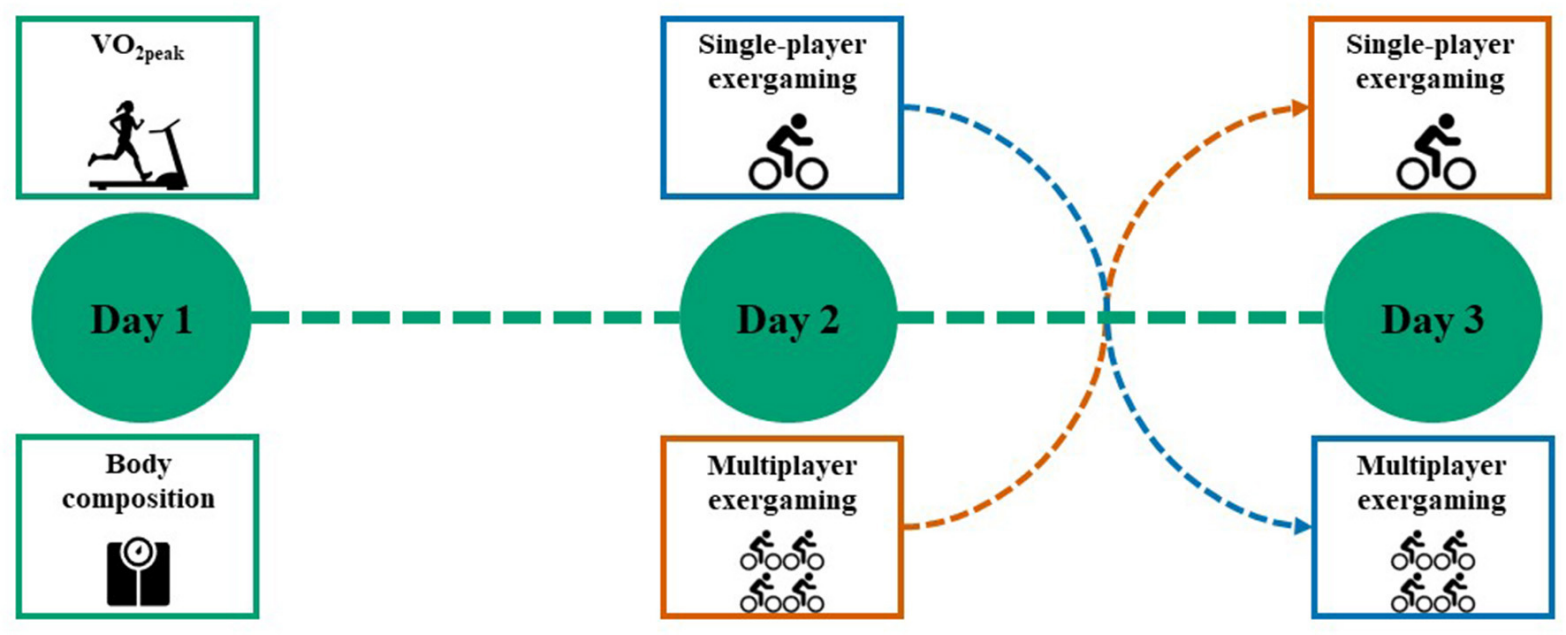
Overview of study outline. All participants completed three assessment days, day 1 including: peak oxygen uptake (V°O_2peak_) and body composition measurements. On days 2 and 3, participants completed a single-player and a multiplayer exergaming session in a randomized crossover fashion.

This randomized crossover trial was undertaken at the Norwegian University of Science and Technology (NTNU) and St Olav's University Hospital in Trondheim, Norway. We conducted three assessment sessions on separate days (Figure 1). We assessed peak oxygen uptake (V°O_2peak_) and body composition on the first day of testing. Participants completed, in random order, two 35-min exergaming sessions playing the Pedal Tanks exergame on the other 2 days: one session was a single-player session and the other a multiplayer session (described below). The Norwegian Data Protection Authority (NSD) approved the study protocol.

### Participants

We advertised the study via social media platforms, the web pages of St. Olav's University Hospital and NTNU, and posters in local fitness centers. To be included in the study, participants had to be healthy, aged between 10 and 30 years old, and able to ride a bicycle ergometer for up to 45 min. We excluded participants with known cardiovascular or metabolic diseases. Before inclusion, all eligible participants signed a written informed consent form.

### The Pedal Tanks Exergame

**Figure 2:**
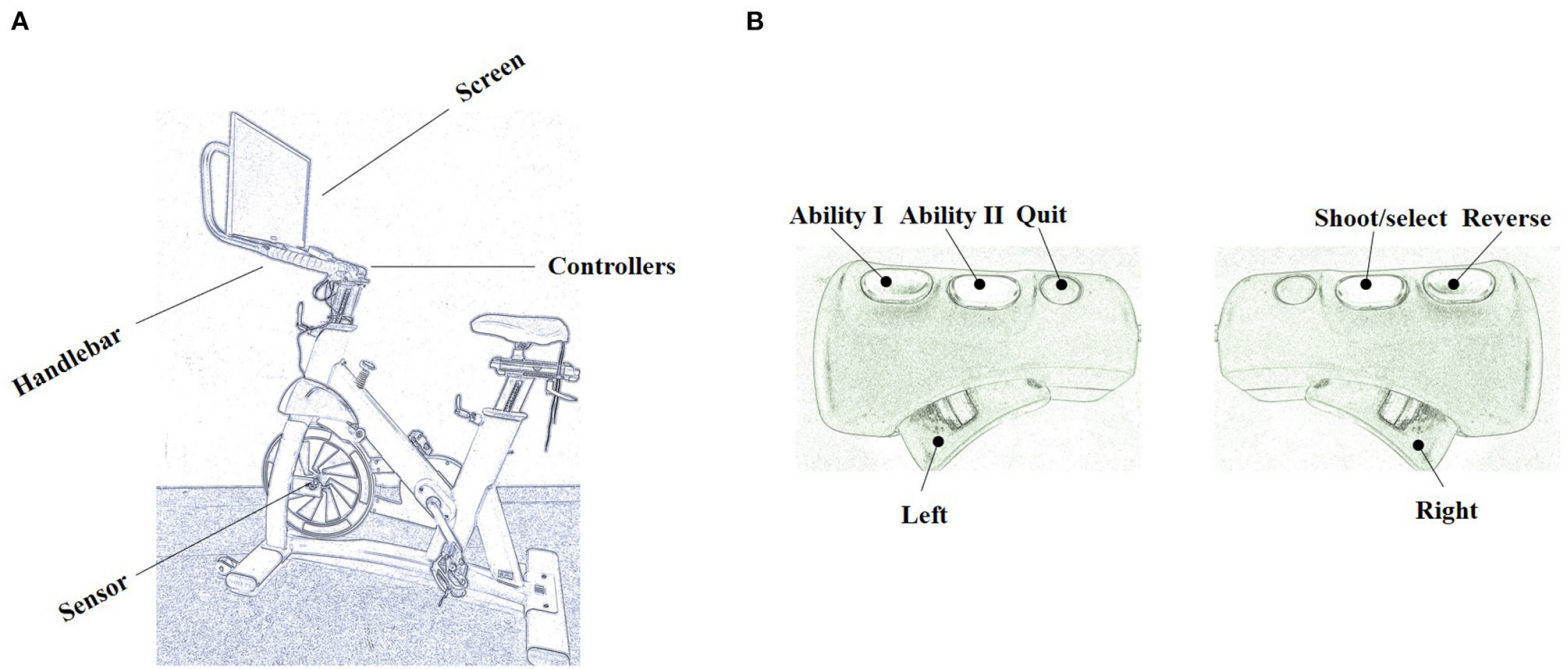
Illustration of the exergaming platform **(A)** with controllers **(B)** used for the present study. This figure was created with pencilsketch.imageonline.co.

The Pedal Tanks exergame is the most frequently used exergame on the Playpulse cycling exergaming platform. In the game, players control actions and steering using buttons on the handlebar, whereas a sensor on the front wheel senses forward propulsion from the pedals generating movement in the game (Figure 2). Pedal Tanks is an online multiplayer arena game with the main objective of capturing the other team's flag and returning it to the team's base (Hagen et al., 2015). Pedal Tanks can be played with up to four players (humans or computer-generated). We used four Playpulse exergaming bicycles for this study, allowing up to four simultaneous human players. Each game is played for a set number of rounds, which terminate once one team captures the other team's flag, or the set timer runs out. Pedal Tanks includes short breaks after each round and game. In Pedal Tanks, players earn experience points during each round, with 1 experience point for every kill, 1 for every assist to a kill, 3 for every flag capture, 10 for every finished round, and an additional 3 for every won round.

Participants played the two exergaming sessions in two modes: single-player and multiplayer. Participants played with one computer-generated teammate and two computer-generated opponents in the single-player session. To balance the participants' skills and the challenges in Pedal Tanks, the participants began the single-player session playing at the lowest of six different difficulty levels. If the participants won the game, the difficulty level increased. In contrast, if the participants lost, the difficulty level decreased or remained at the lowest difficulty level. Participants played together with three other human players for the multiplayer mode, one on the same team and two on the opposing team. Both modes were played for best out of five rounds with a maximum of 5 min per round.

### Outcomes

#### Cardiopulmonary Exercise Testing

Using a maximal incremental exercise test on a treadmill (Woodway, Waukesha, WI) we assessed maximal oxygen uptake (V°O_2max_) and maximum heart rate (HR_max_). After a 10-min warm-up at a self-selected speed and inclination (moderate intensity), participants began the incremental test using the same intensity as they finished the warm-up. Speed/incline increased each minute by 1 km·h^−1^/2% until voluntary exhaustion. We measured gas exchange using MetaLyzer IIIB (Cortex, Leipzig, Germany) throughout the test. Previous data from our lab demonstrated a 1.6 mL·kg^−1^·min^−1^ test-retest repeatability coefficient using the MetaLyzer (Letnes et al., 2020). We calculated V°O_2max_ as the average of the three highest consecutive 10-s values. In addition to voluntary exhaustion, secondary criteria for attainment of V°O_2max_ was a respiratory exchange ratio (RER) ≥ 1.13 and ≥ 96% of age-predicted maximal heart rate (Wagner et al., 2020). Since 60% (*n* = 11) of the participants did not fulfill secondary exhaustion criteria, we use the term peak oxygen uptake (V°O_2peak_). Participants wore heart rate (HR) monitors (H10, Polar Electro Oy, Kempele, Finland) during the incremental test. We report HR_max_ as the peak HR observed during the test.

#### Body Composition

We estimated body mass and percentage body fat using multifrequency bioelectrical impedance (BIA, InBody 770, BioSpace, Seoul, South Korea). We asked the participants to empty their bladder before the BIA assessments since the body composition equations are based on a constant hydration status (Kyle et al., 2004).

#### Exergaming Intensity

To assess exergaming intensity, we measured HR and oxygen uptake (V°O_2_) for the last 20 min of both exergaming sessions, and report both average and peak values. Peak HR and peak V°O_2_ during exergaming were the highest observed 30-s average during the session. In addition, we also express V°O_2_ relative to peak values obtained in the maximal incremental test and report time at or above vigorous (≥64% V°O_2peak_) and high intensity (>75% V°O_2peak_) (Garber et al., 2011; Hawley et al., 2014).

#### Perceptual Responses During Exergaming

We used three separate questionnaires, administered in a random order immediately after each exergaming session, to assess perceived recalled enjoyment, pleasure, and exertion. For enjoyment, we used the total score from the 18-question Physical Activity Enjoyment Scale (PACES), scored on a 1–7 Likert Scale (Kendzierski and DeCarlo, 1991). For pleasure, we used the rating on the 11-point Feeling Scale (Hardy and Rejeski, 1989). The Feeling Scale ranges from −5 (very bad) to +5 (very good). We recorded the rating of perceived exertion (RPE) as the score on the modified 0–10 Borg Scale (Foster et al., 2001).

#### Exergaming Statistics

We obtained exergaming statistics to investigate how specific characteristics of the Pedal Tanks exergame influenced physiological and perceptual responses. From the obtained exergaming statistics, we used the number of rounds played, the percentage of wins in the game, the ratio between kills and deaths, the number of flag captures, and experience points gained.

### Statistical Analysis

Due to the study's exploratory nature, we did not perform a sample size calculation. We used a paired-samples *t*-test to compare the differences in physiological and perceptual responses between the single-player and multiplayer modes. We detected five outliers that were more than 1.5 box-lengths from the edge of the box in a boxplot. We detected three outliers for peak V°O_2_, and one outlier for average V°O_2_ and time at or above vigorous intensity, respectively. However, since inspection revealed that these outliers were not extreme, we kept them in the analyses. Outcome variables were checked for normality via visual inspection of Q-Q plots. We performed simple regression analyses to investigate the relationship between exergaming intensity and perceptual responses, and to assess whether certain exergaming statistics and participant characteristics influenced the attained exercise intensity and the perceptual responses. Average and peak relative V°O_2_ during exergaming, minutes at or above vigorous intensity, minutes with high intensity, perceived enjoyment (PACES score), pleasure (the Feeling Scale score), and RPE were all used as dependent variables. Independent variables were V°O_2peak_ from the incremental test, BMI, body fat percentage, exergaming statistics, perceived enjoyment, pleasure, and RPE. In addition, all independent variables that displayed a significance level of *p* < 0.20 in the simple regression model were entered into a multiple regression model. Since no multiple regression model had more than one variable displaying statistical significance, we only report the results from the simple regression analyses. We performed all data analyses using IBM SPSS 27.0 for Windows (Chicago, IL, United States) and set the significance level at <0.05.

## Results

### Physiological Responses

**Figure 3:**
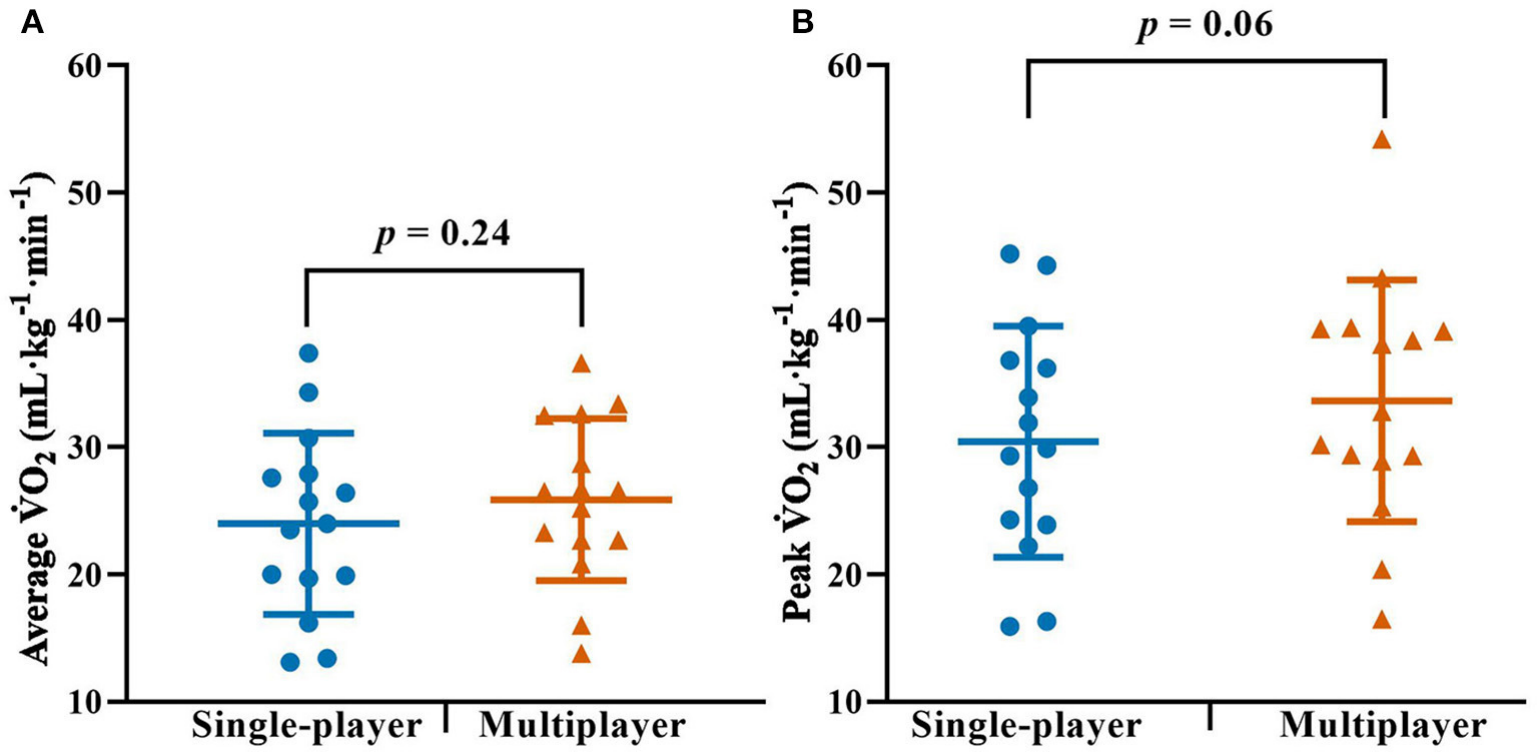
Oxygen uptake during single- and multiplayer exergaming. **(A)** Average oxygen uptake (V°O_2_) and **(B)** peak V°O_2_ during the single- (blue circles) and multiplayer (vermillion triangles) exergaming sessions. Individual data with group means and SD are displayed. *P*-values are from the paired samples *t* test.

**Figure 4:**
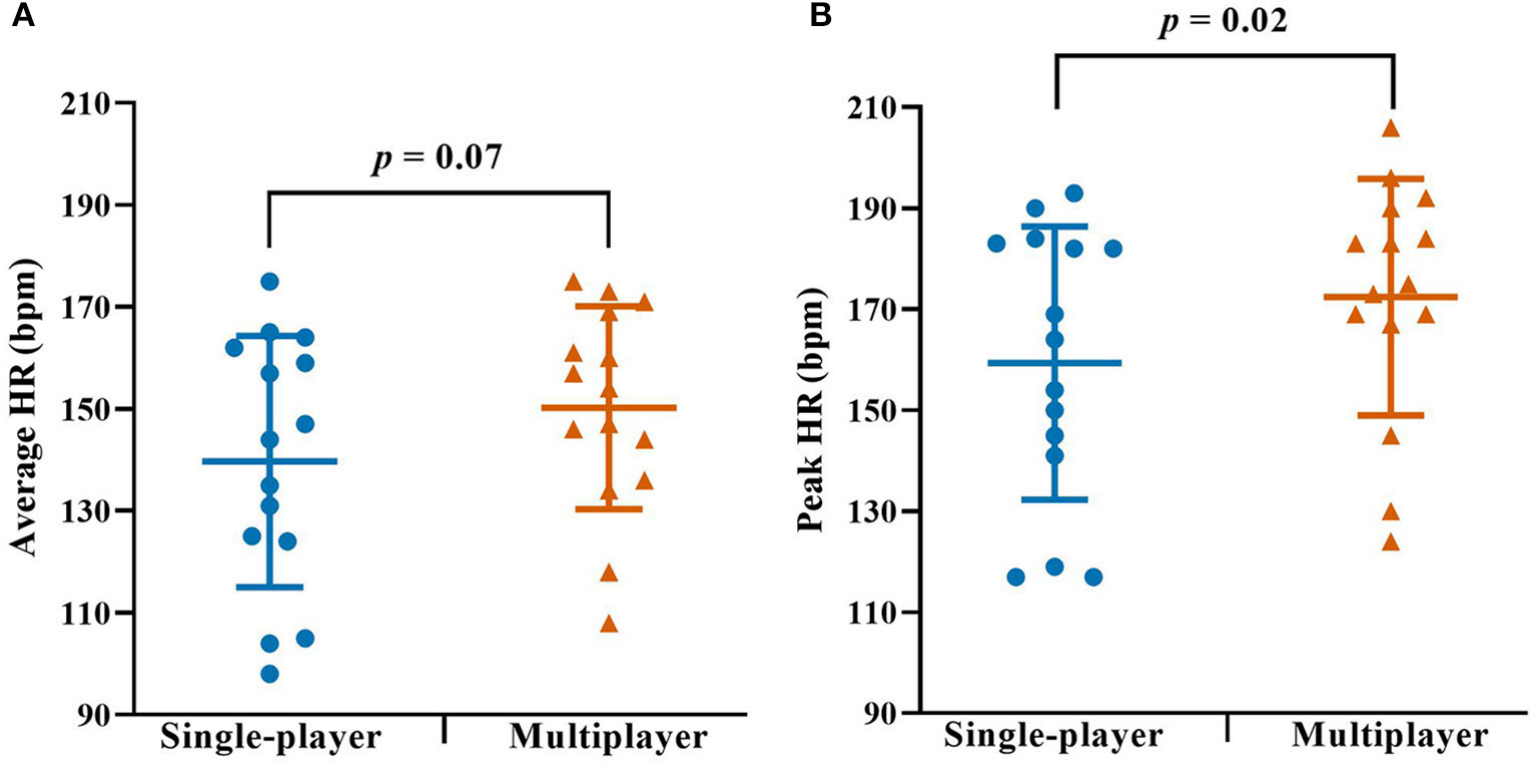
Heart rate during single- and multiplayer exergaming. **(A)** Average heart rate (HR) and **(B)** peak HR during the single- (blue circles) and multiplayer (vermillion triangles) exergaming sessions. Individual data with group means and SD are displayed. *P*-values are from the paired samples t-test.

Table 1 shows the characteristics of the 15 included individuals. The peak and average V°O_2_ during multiplayer exergaming was 25.9 and 33.6 mL·kg^−1^·min^−^, respectively (Table 2, Figure 3). For single-player exergaming, the corresponding values were 24.0 and 30.4 mL·kg^−1^·min^−1^, with no observed difference between the two modes (*p* = 0.06 for peak and *p* = 0.24 for average) (Table 2, Figure 3). With a difference of 13 bpm (95% CI: 2–24, *p* = 0.02), HR_peak_ was significantly higher during multiplayer vs. single-player exergaming. The difference in average HR between the two modes was not statistically significant (*p* = 0.07) (Table 2, Figure 4). For the 20-min exergaming sessions, time spent at or above vigorous intensity was 7.2 min during multiplayer exergaming vs. 4.5 min during single-player exergaming, with no difference between modes (*p* = 0.16) (Table 2). Participants spent on average 3.5 and 1.5 min with a high exercise intensity during multiplayer and single-player exergaming, respectively, with no difference in time between the two modes of exergaming (*p* = 0.11) (Table 2).

**Table 1:** Participant characteristics.

	**All (*n* = 15)**	**Male (*n* = 10)**	**Female (*n* = 5)**
Age	23 ± 7	21 ± 7	27 ± 2
BMI (kg·m^2^)	21.9 ± 3.6	22.1 ± 4.4	21.4 ± 1.0
Bodyfat (%)	24.3 ± 9.4	20.8 ± 9.2	31.2 ± 5.3
V°O_2peak_ (mL·kg^−1^·min^−1^)	46.4 ± 8.9	48.5 ± 9.6	42.2 ± 6.3
HR_max_	196 ± 9	198 ± 10	193 ± 8
Missing data	1 (7%)	1 (10%)	0 (0%)
RER at V°O_2peak_	1.10 ± 0.07	1.10 ± 0.06	1.12 ± 0.09

*Data are mean ± SD or N (%). Body mass index (BMI), peak oxygen uptake (V°O_2peak_), maximum heart rate (HR_max_), respiratory exchange ratio (RER)*.

**Table 2:** Physiological and perceptual responses to single- and multiplayer exergaming.

		**Multiplayer**	**Single-player**	**Difference**	
**Outcome**	***N***	**Mean ±SD**	**Mean ±SD**	**Estimate (95% CI)**	***P***
**Oxygen uptake**
Average V°O_2_ (mL·kg^−1^·min^−1^)	15	25.9 ± 6.4	24.0 ± 7.1	1.9 (−1.4 to 5.2)	0.24
Peak V°O_2_ (mL·kg^−1^·min^−1^)	15	33.6 ± 9.5	30.4 ± 9.1	3.2 (−0.2 to 6.6)	0.06
**Heart rate**
Average HR (bpm)	15	150 ± 20	140 ± 25	11 (−1 to 22)	0.07
Peak HR (bpm)	15	172 ± 23	159 ± 27	13 (2 to 24)	0.02
At or above vigorous intensity (min)	15	7.2 ± 6.6	4.5 ± 6.2	2.7 (−1.1 to 6.5)	0.16
High intensity (min)	15	3.5 ± 4.1	1.5 ± 3.3	2.0 (−0.5 to 4.4)	0.11
**Perceptual responses**
PACES (score)	14	106 ± 10	105 ± 11	1 (−6 to 8)	0.82
RPE	14	3.7 ± 2.0	3.3 ± 1.8	0.4 (−0.7 to 1.5)	0.46
Feeling scale	14	4.0 ± 0.9	3.6 ± 1.5	0.4 (−0.3 to 1.1)	0.29

*Mean ± SD are descriptive data. The effect estimate is from the paired samples t-test and displayed as mean (95% CI). Oxygen uptake (V°O_2_), heart rate (HR), beats per minute (bpm) physical activity enjoyment scale (PACES), rate of perceived exertion (RPE)*.

### Perceptual Responses

There were no differences in perceived enjoyment, pleasure, or exertion between single- and multiplayer exergaming (*p* = 0.29–0.82) (Table 2).

### Relationship Between Exergaming Statistics, Physiological, and Perceptual Responses

The simple regression model for exercise intensity revealed that BMI was the only variable that displayed statistical significance. BMI could significantly predict average relative V°O_2_ (*R*^2^ = 0.17, *p* = 0.03), peak relative V°O_2_ (*R*^2^ = 0.17, *p* = 0.02), and time spent at or above vigorous exercise intensity (*R*^2^ = 0.17, *p* = 0.02) (Table 3). BMI, body fat percentage, and V°O_2peak_ could independently predict RPE during the exergaming session (Table 4). Only the number of rounds played during the exergaming session could predict the score on the Feeling Scale (Table 4). No independent variables could significantly predict time spent with a high exercise intensity or PACES score (Tables 3, 4).

**Table 3:** Regression analyses for average oxygen uptake during exergaming (V°O2_avgEXG_), peak oxygen uptake during exergaming (V°O_2peakEXG_), minutes at or above vigorous exercise intensity (vigorous intensity), and minutes with high intensity (high intensity).

	**V°O**_**2avgEXG**_			**V°O**_**2peakEXG**_			**Vigorous intensity**			**High intensity**		
**Variable**	**B**	**R^**2**^**	***P***	**B**	**R^**2**^**	***P***	**B**	**R^**2**^**	***P***	**B**	**R^**2**^**	***P***
BMI	−1.53	0.17	0.03	−1.93	0.17	0.02	−0.74	0.17	0.02	−0.33	0.09	0.10
Body fat, %	−0.22	0.02	0.43	−0.46	0.07	0.17	−0.10	0.02	0.46	−0.05	0.01	0.57
V°O_2peak_	−0.31	0.04	0.28	−0.11	0.00	0.76	−0.15	0.04	0.27	−0.06	0.02	0.48
RER	5.09	0.00	0.89	−28.87	0.01	0.62	6.00	0.00	0.74	3.33	0.00	0.75
Age-predicted HR	0.69	0.05	0.25	0.83	0.03	0.36	0.11	0.03	0.36	0.03	0.00	0.87
Enjoyment	0.19	0.02	0.46	0.33	0.04	0.29	0.09	0.02	0.45	0.03	0.01	0.68
Pleasure	0.62	0.00	0.88	2.01	0.02	0.45	0.19	0.00	0.86	0.15	0.00	0.80
XP	0.02	0.02	0.43	0.04	0.04	0.29	0.02	0.04	0.30	0.01	0.07	0.17
Winning	−0.02	0.00	0.87	0.00	0.00	1.00	−0.01	0.00	0.78	−0.01	0.01	0.59
Flag captures	0.93	0.03	0.38	1.63	0.06	0.22	0.13	0.00	0.79	−0.08	0.00	0.78
Kill/Death	2.43	0.03	0.41	5.39	0.08	0.14	1.37	0.04	0.33	0.34	0.01	0.67
Rounds	1.00	0.02	0.51	1.10	0.01	0.56	0.28	0.01	0.70	0.36	0.03	0.40

*Unstandardized coefficient beta (B), coefficient of determination (R^2^). Body mass index (BMI), percentage of body fat (body fat, %) peak relative oxygen uptake (V°O_2peak_), heart rate (HR), experience points gained during exergaming (XP), winning percentage during exergaming (winning), flag captures during exergaming (flag captures), the ratio between kills and death during exergaming (Kill/Death), number of played exergaming rounds (rounds)*.

**Table 4:** Regression analyses for enjoyment, pleasure, and rating of perceived exertion (RPE) during exergaming.

	**Enjoyment**			**Pleasure**			**RPE**		
**Variable**	**B**	**R^**2**^**	***P***	**B**	**R^**2**^**	***P***	**B**	**R^**2**^**	***P***
BMI	−0.36	0.01	0.55	0.01	0.00	0.87	−0.21	0.14	0.04
Body fat, %	0.06	0.00	0.78	−0.03	0.04	0.31	−0.11	0.29	0.003
V°O_2peak_	−0.26	0.05	0.24	0.04	0.09	0.12	0.09	0.19	0.02
RPE	0.01	0.00	0.99	0.08	0.02	0.52	-	-	-
XP	0.02	0.04	0.33	0.01	0.10	0.11	0.00	0.04	0.29
Winning	0.04	0.01	0.61	0.00	0.00	0.82	0.02	0.09	0.12
Flag captures	0.93	0.03	0.38	0.06	0.01	0.61	0.04	0.00	0.85
Kill/Death	0.75	0.00	0.76	0.07	0.00	0.82	0.72	0.11	0.09
Rounds	1.22	0.04	0.29	0.32	0.21	0.01	0.09	0.01	0.66

*Unstandardized coefficient beta (B), coefficient of determination (R^2^). Body mass index (BMI), percentage of body fat (body fat, %) peak relative oxygen uptake (V°O_2peak_), experience points gained during exergaming (XP), winning percentage during exergaming (winning), flag captures during exergaming (flag captures), the ratio between kills and death during exergaming (Kill/Death), number of played exergaming rounds (rounds)*.

## Discussion

In this randomized crossover trial, apart from a higher peak HR, there were no statistically significant differences in the physiological and perceptual responses when playing a cycling exergame with other human co-players, compared with when playing alone with computer-generated players. However, although not statistically significant, our findings indicate both a higher average HR and peak V°O_2_ in multiplayer vs. single-player exergaming. Exergaming with others may therefore be a superior exercise alternative compared with exergaming alone.

### Physiological Responses

To our knowledge, this is the first study to compare exercise intensity measured as both HR and V°O_2_ in single- vs. multiplayer exergaming. Thus, our finding that peak but not average HR was higher in multiplayer is novel. However, we found no difference in either average or peak V°O_2_ between the two gaming modes. Previous findings on exercise intensity when exergaming alone compared with when playing with others are mixed. Similar to our findings on average HR and average and peak V°O_2_, there were no differences in estimated energy expenditure or exercise intensity using accelerometry and/or HR between single- and multiplayer exergaming for three different exergames (Peng and Crouse, 2013; Mackintosh et al., 2016; McDonough et al., 2019). However, our finding showing significantly higher peak HR during multiplayer exergaming is in line with the findings by Gorsic et al. (2020). They reported a higher exercise intensity, estimated using an arm accelerometer when the participants played an arm exergame against a human opponent compared with when they played against a computer-generated opponent (Gorsic et al., 2020). An interesting finding in our study is that HR but not V°O_2_ differed between single and multiplayer exergaming. We have previously shown that HR overestimates exercise intensity during exergaming and suggested that V°O_2_ should be used (Berg and Moholdt, 2020). The significant difference in peak HR but not peak V°O_2_ indicates that this overestimation is higher in multiplayer vs. single-player exergaming. A recent review demonstrated an increase in HR when playing regular digital games with a competitive element (Krarup and Krarup, 2020). Therefore, we speculate that an increase in the competitive element when playing against human vs. computer-generated opponents explain part of the discrepancy between peak HR and V°O_2_ observed in the present study. However, potential differences in the competitive element when playing with human vs. computer-generated opponents should be addressed in future studies. Furthermore, although the difference in peak V°O_2_ was not statistically different, there was a numerical difference between the two modes (30.4 vs. 33.6 mL·kg^−1^· min ^−1^).

### Perceptual Responses

Since the presence of a human co-player is an essential factor for player enjoyment in digital gaming, (Gajadhar et al., 2008) we hypothesized that perceived enjoyment and pleasure would be higher in the multiplayer vs. the single-player condition. However, contrary to our hypothesis, we observed no differences in perceived enjoyment, pleasure, or exertion between the two conditions. Our findings align with a recent study that failed to see differences in perceived interest and enjoyment when playing the Wii^TM^ Boxing Exergame alone or with a human co-player (Mackintosh et al., 2016). We extend these findings by examining an exergame that can improve cardiorespiratory fitness after short-term regular use (Berg et al., 2021). On the other hand, our results contrast the findings of two other studies. Peng and Crouse (Peng and Crouse, 2013) reported that enjoyment after an exergaming session was significantly higher in two different multiplayer modes vs. in single-player. Furthermore, in the study by Gorsic et al. (2020) all participants reported that they preferred multiplayer over single-player exergaming. As with the findings for exercise intensity, the use of different exergames may explain these mixed findings. At first glance, the similar perceptual responses between single- and multiplayer exergaming appear to indicate that the two modes are equal in terms of enjoyment and pleasure. However, on closer inspection, our results indicate that multiplayer exergaming can induce higher exercise intensities without sacrificing player enjoyment and pleasure. Furthermore, despite a higher peak HR in multiplayer, the perceived exertion levels were similar between single-player and multiplayer exergaming. Future studies should explore how single- vs. multiplayer exergaming would affect long-term exergaming adherence and enjoyment.

### Exergaming Data

Besides examining any physiological and perceptual differences between single-player and multiplayer exergaming, we aimed to investigate if any participant or exergaming characteristics could influence the physiological and perceptual responses. BMI was the only significant independent predictor of exercise intensity. Average and peak V°O_2_ during the exergaming sessions were 1.5 and 1.9 percentage points lower for each 1 kg·m^2^ increase in BMI, respectively. Furthermore, for each 1 kg·m^2^ increase in BMI, participants spent 0.7 fewer minutes with or above a vigorous exercise intensity. Lower exercise intensity with a greater BMI contrasts with a previous study utilizing the same exergaming platform (Berg et al., 2021). Also, with BMI explaining only 17% of the variance in these variables, we argue that the relationship between BMI and exercise intensity is of limited practical relevance. Furthermore, a greater BMI and body fat percentage were also associated with a lower RPE. Others have shown that an extended work bout duration was associated with time spent with high exercise intensity, (Naves et al., 2019) therefore, we hypothesized that the number of exergaming rounds played would predict time spent in vigorous or high intensity. However, contrary to our hypothesis, we found no relationship between the number of rounds played (or any other exergaming characteristic) and exercise intensity. Overall, we demonstrate that the potency and appeal of this cycling exergame are unaffected by specific physical attributes of the participants or the performance in the game.

### Limitations

This study has several limitations. Primarily, our findings may have been affected by the relatively small sample size. Several of our measures of exercise intensity had *p-*values close to 0.05, which we believe could have been statistically significant with a larger sample size. On the other hand, we argue that the sample size is sufficient to indicate that there might be some important differences between single- and multiplayer exergaming that could affect long-term adherence and effectiveness, which is worth exploring in a long-term study. Secondly, using a treadmill to assess cardiorespiratory fitness whilst the exergame was played on a bicycle ergometer, could be viewed as a limitation. However, since most individuals can attain a higher V°O_2peak_ on a treadmill vs. a cycle ergometer, (Beltz et al., 2016) we argue that the relative expressions of exercise intensity are more valid when using a treadmill for determination of V°O_2peak_. Besides assessing the long-term effects of single- vs. multiplayer exergaming, future studies should be sufficiently powered to explore any possible sex- and age differences.

## Conclusion

Exergaming with others induced a significantly higher peak HR compared with playing alone, but there were no other statistically significant differences in physiological or perceptual responses between single- and multiplayer exergaming. However, there was a tendency for higher exercise intensity during multiplayer vs. single-player exergaming. Future studies should investigate differences between physical and online co-players and whether single- vs. multiplayer exergaming can affect long-term adherence to exergaming.

## Data Availability Statement

The raw data supporting the conclusions of this article will be made available by the authors, without undue reservation.

## Ethics Statement

Ethical review and approval was not required for the study on human participants in accordance with the local legislation and institutional requirements. The Norwegian Data Protection Authority (NSD) approved the study protocol. The participants provided their written informed consent to participate in the study. For the participants under the age of 16, written informed consent to participate in this study was provided by the participants' legal guardian/next of kin.

## Author Contributions

TM and JB were involved in the study design and verified the underlying data. AS and JB collected the data. AS, AW, and JB analyzed the data. JB wrote the manuscript draft. AS, AW, and TM revised the manuscript. All authors approved the final manuscript.

## Funding

The study was funded by the Liaison Committee between the Central Norway Regional Health Authority (Helse Midt-Norge), Grant No. 17/38297. The funding organization had no role in the design and execution of the study, in the collection, analyses, and interpretation of the data, or in preparing the manuscript.

## Conflict of Interest

AW holds some shares in Playpulse AS, the company that develops the Playpulse exergaming platform used in the present study. However, he does not work or is directly involved with Playpulse AS. Furthermore, Playpulse AS had no role in preparing the manuscript. The remaining authors declare that the research was conducted in the absence of any commercial or financial relationships that could be construed as a potential conflict of interest.

## Publisher's Note

All claims expressed in this article are solely those of the authors and do not necessarily represent those of their affiliated organizations, or those of the publisher, the editors and the reviewers. Any product that may be evaluated in this article, or claim that may be made by its manufacturer, is not guaranteed or endorsed by the publisher.
